# Willingness to participate in geolocation-based research

**DOI:** 10.1371/journal.pone.0278416

**Published:** 2022-12-01

**Authors:** Carlos Ochoa Gómez

**Affiliations:** RECSM, Universitat Pompeu Fabra, Barcelona, Spain; Universita degli Studi di Milano, ITALY

## Abstract

Among the new research possibilities offered by smartphones, collecting geolocation data (e.g., GPS coordinates) holds a prominent position, allowing the investigation of individuals’ mobility with greater precision and less effort than conventional data collection methods. However, geolocation data are still affected by errors (e.g., inaccurate recorded locations) and limitations (e.g., inability to record the purpose of a trip). Sending a survey right in the moment an event of interest is detected using geolocation data can add the missing information, while reducing memory errors that typically affect conventional surveys (sent some days/weeks after). However, the possibilities offered by both geolocation data and in-the-moment surveys triggered by geolocation data are limited by individuals’ willingness to participate. This paper assesses such willingness using a conjoint experiment carried out on a sample of 1,016 members of an opt-in online panel in Spain. The effects on such willingness to accept the conditions offered to participants and their personal characteristics are also studied. The results show that asking panelists to participate in in-the-moment surveys does not negatively affect willingness compared to only sharing geolocation data. However, the conditions offered to panelists for their participation (mainly project duration and incentives) have a strong influence on their willingness. Furthermore, panelists less concerned with privacy and safety, and more experienced in sharing social media content, installing apps and using Google Maps, are more willing to participate. Finally, answers to open questions reveal that the main reason for participating is getting the incentive, while not participating is primarily related to privacy issues.

## 1. Introduction

The advent of the mobile internet and the growing distribution of smartphones have provided researchers with new opportunities to collect data passively [1], that is, without the need for the observed individuals to play an active role in the data collection. Passively collected data offer several advantages over actively collected data (e.g., surveys): they are not affected by memory errors and social desirability, and they decrease the burden on respondents by reducing the need for them to answer questions [2].

Among such opportunities, collecting geolocation data from a sample of individuals’ mobile devices is one of the most promising forms. Geolocation data (e.g., GPS coordinates) can be used to identify individuals’ locations and travel patterns [3] or to detect visits to pre-specified locations using geo-fencing [4]. In some of these applications, geolocation data have proved to be more accurate than travel diaries [5].

Nevertheless, geolocation data suffer from different types of errors that restrict their applicability. For instance, the location coordinates recorded by a smartphone are affected by the limited precision of the different technologies used to geolocate devices [6]. Consequently, geolocation data may fail to determine whether an individual has visited a particular location or not. Additionally, a geolocation tracker cannot gather subjective data.

Sending a survey right in the moment an event of interest is detected using geolocation data, also known as geo-triggering [4], can add/clarify information that is missing/ambiguous, while reducing the memory errors that conventional surveys (sent some days/weeks after) usually suffer from [7]. This is a particular application of in-the-moment surveys [8], in this case triggered by geolocation data. Continuing with the previous example, in-the-moment surveys could confirm whether a particular location was visited and ask travelers (detected using geolocation data) about the purpose of the trip and the satisfaction with the means of transport.

However, the potential benefits of geolocation-based research are limited by individuals’ willingness to participate. Although sharing geolocation data is less burdensome for participants than repeatedly reporting visited locations, it may create privacy and safety concerns [9]. Besides, the set-up process required to share geolocation data (i.e., installing an app and granting permissions to use the device’s sensors) may be received as complex and annoying. If participants are also asked to take part in in-the-moment surveys triggered by geolocation data, they must go through an additional set-up process (e.g., activating some sort of instant notification system [10]) as well as agree to be interrupted when an event of interest is detected. Both may negatively affect willingness to participate further [11].

As a consequence, getting participants for geolocation-based research may be challenging and lead to biases if such participants differ from nonparticipants on the variables of interest [2]. Most research on this topic (see section 2.1) has used samples drawn from online panels (both probabilistic and opt-in), leveraging on the already established trust relationship to get higher participation rates compared to other sources of participants (e.g., ad-hoc recruited samples). However, even in this environment the willingness to participate can be a limiting factor depending on the target population and the features of the geolocation-based activity.

The main goals of this paper are to investigate the extent to which members of an opt-in online panel are willing to participate in in-the-moment surveys triggered by geolocation data, and how this willingness is affected by the attributes of such surveys and the characteristics of the participants. Willingness to share geolocation data is also studied for comparison purposes.

More precisely, two geolocation-based research activities were considered:

1. sharing geolocation data in a continuous way for an unspecified purpose. The collected data are stored by the fieldwork company to be used at its convenience.
2. sharing geolocation data in a continuous way for the specific purpose of being invited to in-the-moment surveys. The collected data are not stored but used only to detect events of interest.

A conjoint experiment was carried out on a sample of participants from the Netquest opt-in online panel in Spain, including both activities. This allowed assessing the additional effect on the willingness to share geolocation data of asking panelists to participate in in-the-moment surveys. The second activity has not been researched yet, whereas the first has never been researched using a technique (choice-based conjoint analysis) that allows predicting the expected willingness to participate for any combination of the different attributes that may change in a geolocation-based activity.

## 2. Background

### 2.1. Geolocation data

Gathering geolocation data started to raise interest among researchers when the first GPS commercial devices (e.g., car navigation devices) were marketed in the 2000s. In 2007, the French National Travel Survey was one of the first studies exploring participants’ willingness to share their mobility by means of a GPS receiver [12]: 30% of participants recruited face-to-face agreed to do so (35% if the GPS device could be turned off).

Moreover, interest in geolocation data rapidly grew when smartphones became popular in the early 2010s. Since then, several authors have explored both the willingness to share geolocation data and the actual participation rates under different conditions. Although some of these studies used offline recruited samples [13, 14] or online convenience samples [15], most of the existing research has focused on online panels.

Some studies have focused on one-time capture of geolocation coordinates, finding levels of stated willingness between 30% and 37% [1, 16], and actual completion rates between 20% and 30% [15, 17].

However, most of the substantive research questions that can be better answered using geolocation data (compared to conventional methods) require continuous gathering, which involves installing a tracking application on participants’ smartphones and maintaining its functionality. There has been quite some research over the last few years on both the stated willingness to share geolocation data continuously and the actual participation in such projects. For instance, Keusch and colleagues [2] explored the willingness to install a passive data tracking app (including geolocation data, among others) in an opt-in online panel in Germany: 35% of participants declared they would accept. Revilla and colleagues [18] assessed the willlingness to share geolocation data among Netquest’s panelists in two European and five Latin American countries: 19% to 37% stated they would accept (43% to 61% considering also those who would probably accept). Using the panel from this same company in Spain, Revilla and colleagues [9] assessed the willingness to share geolocation data offering a specific incentive (30 points, almost equivalent to the reward offered for a 25-minute survey) but without indicating a period of time: the willingness was 21%.

As for actual participation, Scherpenzeel [19] reported a study developed on the LISS Mobile Mobility Panel (a probabilistic online panel in the Netherlands), in which panelists with smartphones were recruited to provide geolocation data. Of those who completed the invitation survey (75% of invitees), 37% were willing to participate and 30% (81% of those willing) downloaded the app and provided data for at least one day. Elevelt and colleagues [20] asked a sample of participants from the same panel to share geolocation data in the context of a time use survey that previously required the installation of an app: 69.5% of the participants who completed the pre-questionnaire to take part in the survey also shared geolocation data.

All in all, the willingness to share geolocation data without any additional activity involved usually lies between 20% and 50%, and the differences between stated willingness and actual participation are small in general.

Moreover, many of these studies explored the impact of personal characteristics on the willingness to participate. Some characteristics have been found to positively affect the willingness consistently across several studies: (1) more experience using a smartphone [2, 20] and, specifically, geolocation features [1, 13], (2) liking to share one’s personal life and/or using social networks [9, 13], (3) trust in anonymity of surveys [1, 9], and (4) low privacy and/or security concerns [2, 9, 16]. For others, the effect is only reported in one study: high income, having a computer in the household and having more cars [12], high education [16], having used shopping or travel discount cards [13], being introverted [20], and liking answering surveys and having more experience participating in panel surveys [9].

Finally, a third group of characteristics have produced contradictory results across different studies: (1) age (positive effect for younger people in [12, 13]; negative in [1]), (2) gender (positive effect for males in [12]; negative in [16]) and (3) larger household sizes (positive effect in [13]; negative in [12]).

Much less research is available regarding the potential effects of the conditions offered to participants when asked to share their geolocation. Revilla and colleagues [9, 18] observed lower willingness to participate when the request to share geolocation data did not specify the incentive offered. Keush and colleagues [2] found that incentives strongly influenced willingness, but so did the length of data collection period, the option to switch off the app, and the sponsor of the research (also supported by [1]). However, this research was not specific to geolocation data, only two lengths of the collection period were studied (one and six months), and only a fixed level of incentive was offered.

### 2.2. In-the-moment surveys

In-the-moment surveys have been widely used both offline (e.g., airline passengers satisfaction surveys) and online (e.g., pop-up surveys asking to evaluate an online purchase). Furthermore, the Ecological Momentary Assessment (EMA) [21], a popular research technique in the field of psychology, also uses the idea of surveying people at specific times, minimizing the problem of retrospective memory biases. Participants in EMA studies are sent alarms (e.g., through smartphone apps) asking to report their thoughts, feelings, behaviors, and/or environment in the moment they are asked. Willingness to participate in EMA studies have been found to be high overall, around 89%, depending on the study design features [22].

However, there is very limited literature about in-the-moment surveys triggered by passive data. Although EMA shares some features with this type of surveys, the differences between both methods limit a direct comparison. In particular, the invitations to EMA studies are usually done repeatedly and following a time schedule decided in advance or are implemented at (semi)random, whereas the ones for in-the-moment surveys triggered by passive data are related to the detection of events of interest.

Nevertheless, a few exceptions exist. In-the-moment surveys triggered by geolocation data have occasionally been used in social research, for example to study the respondents’ access to job centers [23], and in commercial research, to evaluate consumers’ exposure to advertisement campaigns, participation in recreational events, or access to health services [4]. Evidence from three commercial research studies developed by Ipsos (www.ipsos.com) shows that barriers to participation in geo-triggered surveys are high, not only due to limited willingness to participate, but because participants have to go through many stages, each one offering opportunities to drop out of the study [24]. Overall, in the three case studies considered, only a fraction of the samples ranging between 0.3% and 3% took part in the in-the-moment survey [4].

Besides such case studies, no formal research has been developed on the willingness to participate in this type of survey. However, Ochoa and Revilla [11] explored the willingness to participate in in-the-moment surveys triggered by another type of passive data: metered data, i.e., data obtained through a tracking application (called a “meter”) installed by the participants on one or more of their browsing devices that registers at least the URLs of the webpages visited [25]. The authors found high levels of willingness for in-the-moment surveys triggered by online behaviors in a metered panel in Spain, ranging from 69% to 95%, depending on the conditions offered to participants. However, there are substantial differences between geolocation and metered data that could lead to different levels of willingness. Besides, Ochoa and Revilla [11] focused on participants who already shared metered data which prevented assessment of the influence of having to share passive data on the overall willingness to participate in in-the-moment surveys.

## 3. Research questions, hypotheses, and contribution

The main purpose of this study is to assess the willingness of panelists to participate in in-the-moment surveys triggered by geolocation data. To that end, the willingness to share geolocation data is also studied in the same sample of panelists. By doing this, the additional effect of asking panelists to participate in in-the-moment surveys can be measured.

In line with Ochoa and Revilla [11], the following research questions and hypotheses are proposed.

*RQ1 –*To what extent are panelists willing to share geolocation data (*RQ1a*) and to participate in in-the-moment surveys triggered by geolocation data (*RQ1b*) under different conditions?

The expected levels of willingness to share geolocation data are similar to those in previous literature, i.e., between 20% and 50% (*H1a*).

As for in-the-moment surveys triggered by geolocation data, at least three factors could affect the willingness. First, answering surveys requires an additional effort compared to only sharing geolocation data, although this effort can be offset by an additional incentive. Second, surveys asking participants about details of their recently visited locations may raise higher privacy concerns. However, geolocation data collected to trigger in-the-moment surveys are not stored, but used only to detect events of interest, which may lessen such concerns. Third, the need to answer surveys within a time limit may interrupt participants’ normal activities, but Ochoa and Revilla [11] found that, for surveys triggered by metered data, the need to answer in the moment barely affected participants’ willingness.

All in all, the willingness to participate in in-the-moment surveys triggered by geolocation data is expected to be similar to the willingness to share such data (*H1b*).

*RQ2* –How do the attributes of geolocation-based research projects influence the willingness to participate?

The following five attributes are considered in addition to the research activity: (1) project duration, (2) geolocation incentive (offered to panelists in return for sharing geolocation data over a period of time), (3) invitation lifetime (maximum time to start the survey after the invitation is sent), (4) survey length, and (5) survey incentive level (incentive offered for participating in in-the-moment surveys compared to a conventional survey). Project duration and geolocation incentive are defined for both geolocation-based activities, while the remaining three attributes are specific to in-the-moment surveys.

Project duration is expected to have a negative influence on the willingness to participate (*H2a*). Since geolocation data may be perceived as sensitive, participants should prefer to share such data the shortest possible time, in line with the results of Keush and colleagues [2]. However, shorter durations prevent panelists from earning more incentives (see section 5.2), which may lead some panelists to prefer longer durations.

Geolocation incentive, defined separately from the specific incentive offered for participating in in-the-moment surveys, is expected to have a positive effect on the willingness to participate (*H2b*).

The three survey-specific attributes are those used by Ochoa and Revilla [11] for in-the-moment surveys triggered by metered data. Everything else being equal, a higher willingness to participate is expected for longer surveys (since they offer higher incentives, see section 5.2) (*H2c*), longer invitation lifetimes (*H2d*) and higher survey incentive levels (*H2e*).

Also, in line with Ochoa and Revilla [11], incentive-related attributes (survey incentive level and geolocation incentive) are expected to produce the largest effects both in sharing geolocation activities (*H2f*) and in-the-moment surveys (*H2g*).

Finally, the location visited by panelists was not included as an attribute in this study since there are too many possible locations (see section 5.2). Instead, participants were asked about their willingness to participate in five specific situations (see section 5.3). Their effect on the willingness is expected to be limited (*H2h*) due to the difficulty for participants to foresee the actual implications of participating in different locations/situations.

*RQ3* –Are there significant differences in the willingness to participate in geolocation-based research among panelists with different profiles in terms of sociodemographic characteristics, personality traits, attitudes/behaviors and panel experience?

To explore differences among participants, similar variables as in Ochoa and Revilla [11] are used. For the sociodemographic characteristics, a higher willingness is expected for males (*H3a*), for younger people (*H3b*), for more educated people (*H3c*) and for those living in smaller households (*H3d*).

The effect of personality traits is explored using the “Big 5” dimensions [26]: a higher willingness is expected for those with high agreeableness (*H3e*) and openness (*H3f*), whereas no significant influence is expected for different levels of consciousness (*H3g*), extraversion (*H3h*) and emotional stability (*H3i*).

As for attitudes/behaviors, higher levels of willingness are expected for those who trust survey privacy (*H3j*) and safety (*H3k*), and frequently share content on social media (*H3l*). In addition to these three variables used by Ochoa and Revilla [11], two variables related to smartphone usage are considered: whether the panelists usually install applications on their smartphones (higher willingness expected if they do; *H3j*) and whether they normally use a particular app (Google Maps) that requires sharing their geolocation (higher willingness expected if they do; *H3n*), since both are needed to participate in geolocation-based research.

Finally, panel experience was measured by means of two variables: number of past participations in panel surveys and experience sharing metered data. A higher willingness is expected for those with a higher number of past participations (*H3o)* and for those already sharing metered data *(H3p)*.

The above hypotheses are defined in line with the literature about sharing geolocation data (see section 2.1) and in-the-moment surveys (see section 2.2). In case of contradiction between the two (e.g., gender), the hypotheses are established based on the literature about sharing geolocation data, since this activity is expected to be more relevant on panelists’ decisions than taking in-the-moment surveys (see *RQ1*).*RQ4* –What are the main reasons stated by the panelists for deciding whether or not to participate?

Open questions are used to collect motivations and concerns in addition to the attributes considered in the conjoint experiment used to answer the first three research questions. According to the literature, incentives are expected to be the most frequently mentioned reason for participating (*H4a*), and privacy concerns for not participating (*H4b*).

By answering these research questions, this paper contributes to the existing knowledge in several ways. First, willingness to participate in in-the-moment web surveys triggered by geolocation data has not been researched yet. Besides contributing to the growing body of literature about willingness to participate in additional research tasks, this study aims to help researchers to evaluate the feasibility of obtaining a sample from an online panel for their geolocation-based research, as well as provide guidance to improve the participation.

Second, although the willingness to share geolocation data has been studied before, this paper delves into the effect of two critical attributes (i.e., project duration and incentive).

Finally, both the willingness to participate and the attributes that influence it are researched using a choice-based conjoint analysis (CBC). This method allows measuring the effect of each attribute level on the willingness to participate, as well as making predictions for specific combinations of such attributes. CBC provides more accurate predictions and better estimates of each attribute’s relevance than alternative methods (e.g., direct questions asking participants if they would participate in these activities) by forcing participants to make a trade-off between multiple desirable and undesirable attributes, as normally occurs in real-life decisions [27, 28]. CBC has already been used to research willingness to participate in various non-survey research tasks [29] and also in in-the-moment surveys triggered by metered data [11]. However, it has never been used to research willingness to participate in geolocation-based research. Keusch and colleagues [2] used vignettes, which is a form of conjoint analysis but not choice based.

## 4. Data

The data were collected in Spain between February-March 2022 in the Netquest (www.netquest.com) opt-in online panel. Netquest panelists regularly participate in surveys rewarded with points that can be redeemed for gifts. The longer the surveys are, the larger the number of points they receive. In addition to taking surveys, some of the panelists are asked to share metered data. In exchange, they earn two additional points per week for each device with the meter installed, up to a maximum of three devices. The panelists invited to join the metered panel are not randomly selected from the survey panel. Instead, those panelists with higher likelihood to accept the meter installation according to an internal predictive algorithm are invited, leading to an installation rate of around 30% in Spain [30].

In this study, the objective was to get a sample of 1,000 panelists who use a smartphone on a daily basis. Quotas for gender, age and education were defined to reproduce the proportions of the online population according to the National Statistics Institute of Spain (www.ine.es). In addition, a quota was set to reproduce approximately the proportion of metered panelists’ surveys that the panel delivers in a typical survey project. This was done because even though metered panelists represent approximately 10% of the full survey panel, they are more active. Thus, they represent a higher proportion of the respondents who complete a particular study. Such proportion was estimated to be 25% on average (i.e., their response rate is approximately 3 times that of non-metered panelists).

From the 2,306 panelists invited to the survey, 1,847 started it (80.1%), and 1,016 were considered valid participants (44.1%), while 461 (20.0%) were discarded without taking the full questionnaire for different reasons: not using a smartphone daily (59), exceeding the quotas (222), not giving their explicit consent to participate (162) and not passing basic anti-fraud checks (18). The remaining 370 (16.0%) dropped out during the survey.

The average age of the valid participants is 46 years. 50% are women. 26% are mid-educated and 34% are highly educated. On average, they have been in the panel for 5.9 years and completed 290 surveys (approximately one per week). Metered panelists represent 28% of the valid participants; those are the most experienced ones (7.1 years in the panel and 359 completed surveys).

## 5. Methods

### 5.1. A conjoint approach

In order to answer *RQ1*, *RQ2* and *RQ3*, participants were asked about their willingness to participate in geolocation-based research using a CBC analysis. CBC estimates causal effects of multiple attributes by asking participants to repeatedly select among different combinations of attribute levels. The analysis of their choices allows estimating the contribution of each attribute level to the decision to participate, which in turn can be used to estimate the expected willingness to participate of each individual for any combination of attribute levels. This information can be used to estimate the average willingness to participate in different scenarios and determine differences among groups of participants.

A complete review of this method is provided by Orme and Chrzan [28], and a description of how it can be adapted to measure the willingness to participate is provided by Ochoa and Revilla [11].

### 5.2. Design of the conjoint experiment

First, participants were shown a general description of the two different geolocation-based activities (sharing geolocation data and in-the-moment surveys triggered by geolocation data) and the different attributes that may change among them. Then the conjoint experiment proposed ten questions showing two possible geolocation-based activities and asked the participants to choose which one they would prefer to participate in.

**Fig 1:**
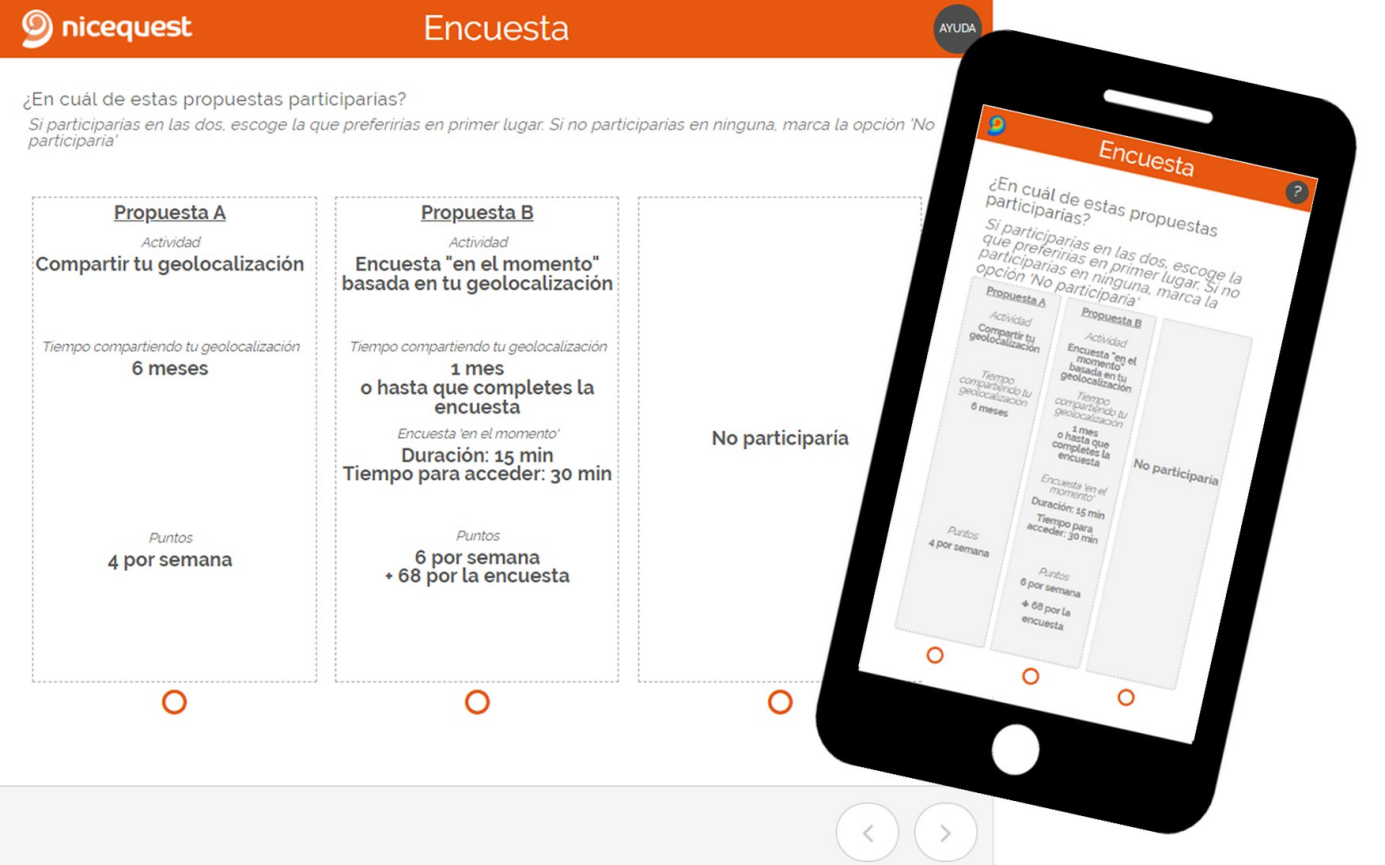
Example of a conjoint question (PC and smartphone views).

Each activity shown was defined as a combination of the research activity and the respective levels of the attributes. The option “I would not participate” was offered too (screenshots available in Fig 1).

The number of questions, attributes, levels, and proposals were chosen to ensure the stability of estimations at an aggregated level [28], while limiting the cognitive burden typically caused by the repetitive nature of the conjoint questions. The combination of attribute levels used in each pair of activities for each question was designed to optimize D-efficiency [31] by means of a proprietary algorithm that follows the general method for constructing efficient choice designs [32]. Further information on the experimental design is available in the supplementary online material (https://osf.io/qt52v).

For each activity and the five attributes included in the conjoint experiment, the following levels were defined:

- Activity: One of the two activities of interest, 1) sharing geolocation data or 2) in-the-moment surveys triggered by geolocation data. Participants were informed that the geolocation data gathered for in-the-moment surveys will only be used to detect visits to locations of interest (not stored).
- Project duration: Previous research has found lower willingness when participants are asked to share mobile passive data for 6 months compared to 1 month [2]. In this study, this range was expanded: 1 week, 1, 3 and 6 months and 1 year. Considering long periods (e.g., 1 year) is needed for in-the-moment surveys, since some locations of interest may be visited only occasionally (e.g., a hospital). The level “indefinite” was also added to assess the effect of not specifying in advance an activity’s duration.
- Geolocation incentive: Both activities included a specific incentive for sharing geolocation data over time. Netquest offered in the past between 2 and 5 points per week to panelists in exchange for sharing geolocation data for specific projects. On that basis, the following levels were defined: 1, 2, 3, 4, 6 and 8 points per week.

For the attributes specific to in-the-moment surveys, the same levels as Ochoa and Revilla [11] were used:

- Survey length: 1, 5, 10, 15 and 20 minutes.
- Invitation lifetime, i.e., the maximum time allowed to start the survey after the invitation is sent: 15, 30 minutes, 1, 2, 3, 6 and 12 hours.
- Survey incentive level: 1, 1.5, 2, 3 and 4 times a conventional survey. Survey incentive levels were not shown as such to participants. Instead, they saw the number of points that they would get for answering the survey. For instance, a conventional 10-minute survey would be rewarded with 12 points according to the existing panel policy; if the incentive level was 1, the participants saw that they would get 12 points, whereas if the incentive level was 2, they saw that they would get (2x12 =) 24 points. This design does not allow measuring the effect of the total number of points on the willingness to participate as it was not the purpose of this research. Only the effect of the incentive level (compared to a conventional survey) is measurable [11].

Finally, three different wording styles were used to present the information about the activities, each one randomly assigned to one third of the sample: (1) a “neutral” style that simply asked respondents if they “would participate” (similar to Ochoa and Revilla [11]), (2) a “commitment” style, putting emphasis on the need for participants to commit to completing the activities, and (3) a “safety” style that reassured participants that their data were safe. Results from this experiment [33] showed that communication style has a significant but relatively small effect on willingness (maximum difference of 7%). In this paper the average results for the three groups are presented.

### 5.3. Full questionnaire

The final questionnaire included up to 38 questions that were asked in an online survey optimized for mobile devices. The average time to complete the questionnaire was 8.8 minutes and the median 7 minutes. An English translation of the full questionnaire and screenshots are available in the supplementary online material (https://osf.io/zjwyd).

Respondents could continue without answering the questions, except those used to control quotas and filter other questions. A warning message was shown to 29 participants who tried to skip a question when multiple questions were presented on the same page. Following the panel’s usual practice, going back was not allowed.

Besides the ten conjoint questions, participants who declared they would participate in in-the-moment surveys in at least one of the proposed scenarios were asked five questions (using 0–6 point scales) about their willingness to participate in these specific situations: (1) Visiting a hospital for a scheduled visit (not urgent), (2) shopping in a supermarket, (3) traveling to a nearby location, (4) visiting a COVID vaccination center, and (5) visiting a bathing area (beach, river, lake). The description of each situation was completed using fixed intermediate values for the survey attributes (survey length = 10 minutes, invitation lifetime = 1.5 hours and survey incentive level = 2.5 times a conventional survey, which resulted in a total incentive of 30 points).

Furthermore, four open questions asked participants about the reasons to participate or not in both geolocation-based activities. Moreover, sociodemographic, personality, and attitudinal/behavioral questions were included to identify differences in the participants’ willingness to participate, as in Ochoa and Revilla [11]. Finally, two yes/no questions were included to ask participants whether they frequently install apps on their smartphones and search for locations on Google Maps (as a representative example of a smartphone activity that requires sharing geolocation).

The number of past participations in panel surveys and whether participants were already sharing metered data were used as measures of the panelists’ experience. These data were not asked in the survey but directly provided by Netquest.

### 5.4. Analysis

The analyses were performed using R 4.1.2. The scripts and the data are available in the supplementary online material.

#### 5.4.1. Choice model

The participants’ answers to the conjoint questions were analyzed using a mixed logit model [34].

Individual attribute-level utilities, as well as the upper-level distribution parameters, were estimated using a Bayesian procedure developed by Allenby [35] that requires estimating the entire probability distribution for each parameter by means of simulations. From the posterior distributions, 60,000 random draws were generated using Markov Chain Monte Carlo [36], but only the last 20,000 were used for estimations, to ensure convergence to the actual distributions. Convergence was assessed by visually inspecting the stability of the estimations using a trace plot. To avoid autocorrelation among draws, only one every tenth draw was saved, resulting in 2,000 posterior draws [37].

Following Orme and Chrzan [28], point estimates, credibility intervals and significant differences were assessed using the posterior draws, using a Bayesian approach. For instance, 95% credibility intervals were estimated computing the 2.5^th^ and 97.5^th^ quantiles of the posterior draws. Similarly, to assess with 95% confidence (5% significance level) if utilities were significant, the proportion of the posterior draws greater than zero was evaluated; if this proportion was less than 0.05/2 or more than 1–0.05/2, the utility was considered significant (equivalent to a two-tail test). The same approach was used to assess significant differences between two utilities and other quantities calculated on them, such as the willingness to participate.

#### 5.4.2. Utilities to assess influence on the willingness to participate

Utilities were first used to answer *RQ2*. Although the interpretation of utilities in a mixed logit model is not straightforward, they are informative about which attribute levels influence willingness the most and in which direction.

Utilities are scaled to sum zero within each attribute and must be interpreted in relative terms. Positive utilities contribute to the willingness above the average, and vice versa. The attribute “none”, associated with the option “I would not participate” shown in each conjoint question, was coded differently. This attribute measures the utility of not participating, assuming implicitly a reference level of zero for participating. The utility of this attribute was used to assess the willingness to participate (see section 5.4.3).

Utilities were also used to evaluate the overall importance of each attribute on participants’ decisions. Relevant attributes are those presenting more variation in utility among levels. The total variation within an attribute (i.e., largest minus smallest utility) over the sum of variations of all the attributes (excluding the “none” attribute) is a popular measure of attribute importance in choice models [37].

#### 5.4.3. Transforming utilities into willingness to participate

Both *RQ1a* and *RQ1b* (willingness to share geolocation data and to participate in in-the-moment surveys, respectively) can be answered by transforming utilities into choice probabilities using the multinomial logit formula. Such choice probability is calculated by summing the total utility of the attribute levels involved in each geolocation-based research activity and comparing the sum to the utility of not participating (measured by the option “I would not participate”).

#### 5.4.4. Differences in willingness to participate

To answer *RQ3*, the average willingness to participate of each group of participants is calculated using their individual utilities.

In order to allow the mixed logit model used in the analysis to better identify differences among groups of participants, covariates were added to the upper-level distributions, allowing the distribution mean to be a linear combination of participants’ characteristics [38]. Since each covariate level doubles the number of utilities to be estimated, which reduces the precision of the estimates, covariates were added to the model in groups. First, an initial baseline analysis was developed including as covariates (1) whether panelists were sharing metered data, (2) communication style and (3) sociodemographic variables (gender, age, education, and household size). The analysis was then repeated, adding only one of the following groups of covariates each time: (1) attitudes/behaviors, (2) personality traits, (3) geolocation usage, and (4) past experience as panelists.

All the covariates were added to the model as categorical variables. To that end, numerical variables were binned into groups (https://osf.io/zjwyd). Each covariate produced 31 coefficients (one for each attribute level in the model) times the number of covariate levels minus one. The willingness to participate was calculated for each covariate level using the simulated posterior draws (see section 5.4.1.); the size of the effects and whether they are significant is also reported. However, to decide if hypotheses are supported, the relevance of the differences is considered instead of just the results of the test of statistical significance [39].

#### 5.4.5. Open questions

Open questions were used to answer *RQ4*. The coding process looked for aspects of geolocation-based research activities that could lead panelists to participate or not, in order to detect whether there were other relevant aspects beyond those considered in the conjoint experiment.

Answers were coded by two native speakers. First, the main coder produced an initial codebook. After that, a secondary coder repeated the process using the same codebook. The intercoder reliability was 93%. The reported results are those of the main coder, after review based on those of the second coder.

Only reasons with more than 5% of mentions are reported. When respondents provided several reasons, all of them are considered.

### 5.5. Ethics

Written consent of all participants was obtained and recorded in the web survey used to gather the data for this study. The text shown to participants to obtain such consent is available as supplementary online material (https://osf.io/zjwyd).

This study is part of a project reviewed and approved by the Institutional Committee for Ethical Review of Projects of the Universitat Pompeu Fabra (CIREP-UPF; https://www.upf.edu/web/cirep; approval number: 135).

## 6. Results

### 6.1. Factors influencing the willingness to participate

#### 6.1.1. Preference among attribute levels

**Fig 2:**
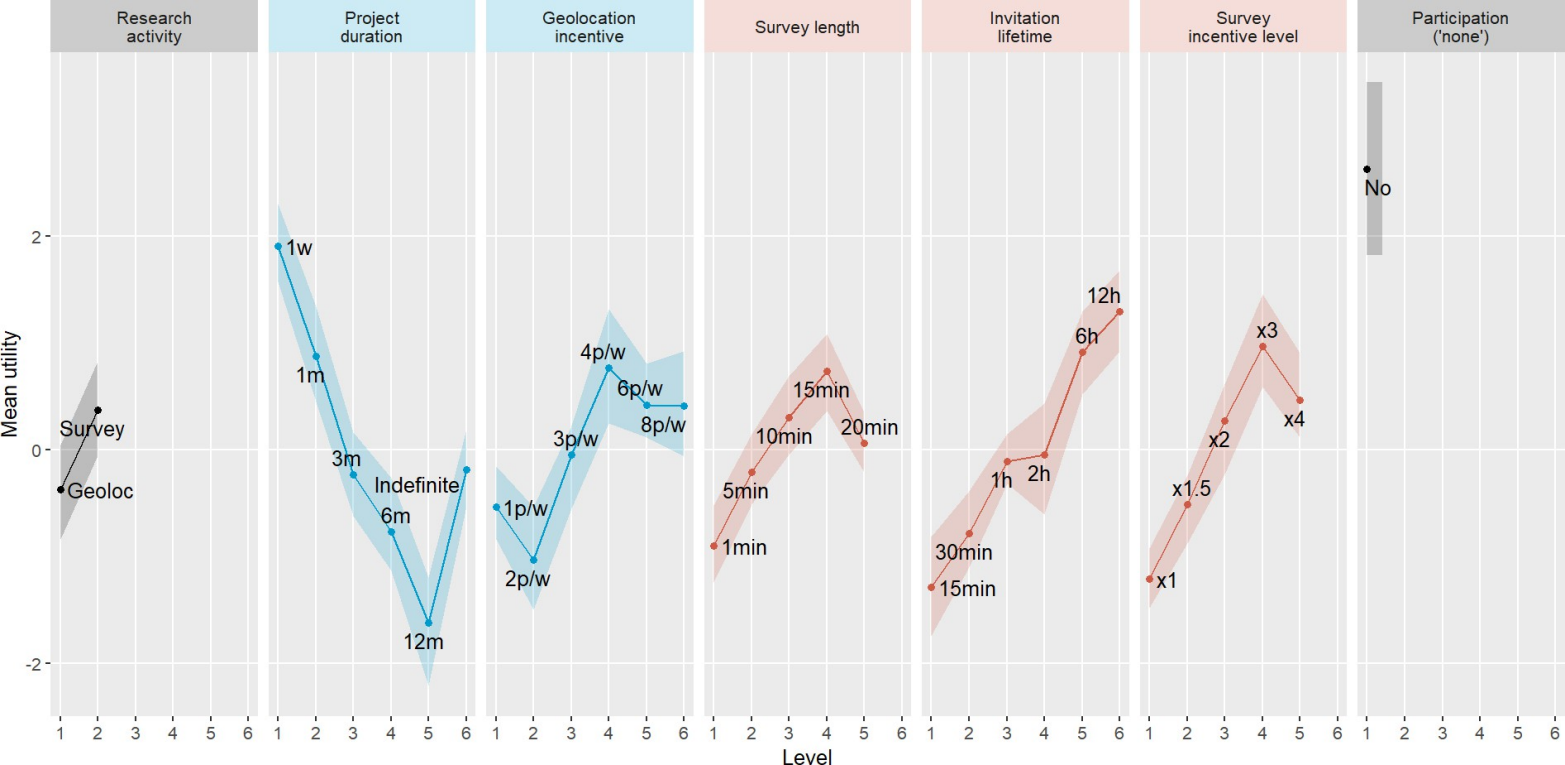
Mean utilities per attribute level and 95% credibility intervals (shadowed bands). Note: Blue subplots = common attributes for both geolocation-based activities; red subplots = specific in-the-moment surveys’ attributes. Grey subplots = rest of the attributes needed to model participants’ choices.

To answer *RQ2*, Fig 2 shows the mean utilities per attribute level (the shadowed band corresponds to a 95% credible interval), revealing how utilities change alongside attribute levels.

First, as expected (*H2a*), the higher the project duration, the lower the utility, indicating a clear preference for sharing geolocation data for shorter periods of time. The level “indefinite” gets an intermediate utility, similar to a project duration of three months. Since geolocation incentives are defined on a weekly basis (i.e., the more weeks sharing data, the more points earned), this result suggests that the participants prefer shorter times over the possibility of receiving more incentives.

The geolocation incentive has a positive effect on the utility in general (support for *H2b*). However, results suggest that participants only differentiate between two levels of incentive:1–2 points per week and 4–6 points per week, with the 3-points level falling in the middle. The differences between these two groups are significant, whereas within groups they are not.

Regarding the in-the-moment surveys’ specific attributes, survey length has a positive influence on the willingness to participate but only up to 15 minutes (partial support for *H2c*). The positive effect of survey length, also found by Ochoa and Revilla [11] for in-the-moment surveys triggered by metered data, may be explained by the fact that longer surveys offer more incentives. In other words, this result suggests that the participants value the higher incentives more than the inconveniences caused by longer surveys (unlike the effect of the project duration). However, contrary to what was found for metered data, when surveys are triggered by geolocation data, participants prefer not to exceed 15 minutes. This could indicate that they expect to have less time available to participate when performing physical activities (e.g., shopping in a supermarket) than online activities (e.g., shopping online).

Furthermore, longer invitation lifetimes are related to higher utilities (support for *H2d*). Participants prefer to have as much time as possible to participate.

As for the survey incentive level, it has also a positive effect on the utility but only until the level “3 times a conventional survey” (partial support for *H2e*). Surprisingly, “4 times a conventional survey” is worse perceived than 3 times. Although this difference is not significant (at 5% level), it may indicate that above 3 times a conventional survey the incentivization does not produce a relevant effect. It is interesting to note that when metered and non-metered panelists are analyzed separately, this effect is not found for metered panelists, who prefer the highest survey incentive level. Since metered panelists have already accepted to share a form of passive data, they may be both more sensitive and less suspicious when offered higher incentive levels. However, further research is needed to explain this effect.

Finally, participants who stated they would participate in in-the-moment surveys under some circumstances (N = 662) were also asked about specific but common situations that could trigger such surveys, using scales from 0 (“For sure, I would not participate”) to 6 (“For sure, I would participate”). Table 1 shows for each situation the mean score, standard deviation, and the proportion of panelists that would participate (score >3). The incidence of each situation among panelists (percentage of participants performing the activity described in each situation) is also included. Participants who reported never experiencing a situation were excluded from the analysis (between 4 and 38 cases per situation).

**Table 1:** Willingness to participate in in-the-moment surveys (0–6 point scales) in five specific situations.

Situation	Incidence (%)	Mean	SD	Percentage answering >3
Shopping in a supermarket	99.4	4.87	1.65	82.8
Visiting a COVID vaccination center	94.6	4.76	1.77	80.0
Travelling to a nearby location	97.9	4.73	1.72	80.7
Visiting a bathing area	94.2	4.67	1.79	79.7
Visiting a hospital for a scheduled visit	96.2	4.62	1.83	78.7

Taking a survey while shopping in a supermarket is the most accepted situation while visiting a hospital for a scheduled visit is the least accepted one, but the differences are minimal (score difference = 0.25, participation difference = 4.1 percentage points). This supports *H2h*. Although the five situations studied represent only a very limited sample of all the potential situations that could trigger in-the-moment surveys, they are quite diverse, suggesting that this factor may not be particularly relevant. However, actual participation may differ from the stated willingness to participate due to practical issues that cannot be foreseen by participants. For instance, it may be easier to complete a survey when visiting a bathing area than after shopping (e.g., while carrying supermarket bags).

#### 6.1.2. Importance of attributes

Attribute-level utilities can be used to estimate its relevance in the participants’ decisions. Table 2 presents the importance for each research activity and attribute.

**Table 2:** Importance of attributes.

Sharing geolocation			
		**Percentile**	
**Attribute**	**Importance (%)**	**2.5** ^**th**^	**97.5** ^**th**^
Project duration	64.3	54.6	74.5
Geolocation incentive	35.7	25.5	45.4
**In-the-moment surveys**			
		**Percentile**	
**Attribute**	**Importance (%)**	**2.5** ^**th**^	**97.5** ^**th**^
Project duration	29.6	24.9	37.0
Invitation lifetime	21.8	17.1	26.9
Survey incentive level	18.4	15.1	22.3
Geolocation incentive	16.4	12.3	22.6
Survey length	13.7	15.1	22.3

When participants need to decide whether to share geolocation data, project duration is clearly more relevant than incentive (64.3% vs. 35.7%; no support for *H2f*). However, when the decision is about participating in in-the-moment surveys, the number of attributes to be considered is consequently extended, leading to a reduced importance of the two common attributes. In this second case, the two incentive-related attributes (geolocation incentive and survey incentive level) become the most relevant ones, with a total importance of 34.8% (18.4+16.4), which brings support for *H2g*. In fact, considering that the panelists’ preference for longer surveys (seen in section 6.1.1.) could also be explained by incentives, the total importance could be even higher.

A possible explanation for this fact may be that the incentives are of limited relevance for activities that are sensitive but require little effort (once the set-up is completed). Conversely, the incentives may become more relevant for activities that are not sensitive but require greater effort, such as answering in-the-moment surveys.

As for the remaining survey attributes, invitation lifetime is more important than survey length, contrary to what Ochoa and Revilla [11] found for surveys triggered by metered data. This suggests that participants perceive the requirement to participate within a time limit as more problematic when surveys are related to physical activities compared to online activities.

### 6.2. Willingness to participate

In order to assess what levels of willingness may be expected for different activities, Table 3 shows the estimated willingness to participate for sharing geolocation data (*RQ1a*) and in-the-moment surveys activities (*RQ1b*) for three different scenarios:

1. **Best scenario**. A scenario consisting of those attribute levels with highest average utilities. For sharing geolocation data that is: project duration = 1 week and geolocation incentive = 4 points/week. In addition, for in-the-moment surveys, the best scenario includes: survey length = 15 minutes, invitation lifetime = 12 hours and survey incentive level = 3 times the equivalent conventional survey.
2. **Worst scenario**. A proposal consisting of those attribute levels with lowest average utilities. For sharing geolocation: project duration = 12 months and geolocation incentive = 2 points/week. In addition, for in-the-moment surveys: survey length = 1 minute, invitation lifetime = 15 minutes and survey incentive level = 1 time the equivalent conventional survey.
3. **Average scenario.** This scenario has been defined differently. First, the average willingness to participate in all the scenarios has been calculated for each participant. Then, the average of the individual average willingness is reported.

**Table 3:** Expected willingness to participate.

			Percentile	
Research activity	Scenario	Mean willingness to participate (%)	5^th^	95^th^
Sharing geolocation	Best	50.1	46.8	53.7
	Average	43.2	41.1	45.1
	Worst	37.6	35.6	39.6
In-the-moment surveys	Best	57.1	55.2	59.3
	Average	47.2	46.6	47.8
	Worst	34.4	32.4	36.2

As expected (*H1a*), the levels of willingness to share geolocation are in line with the literature (43.2% on average). As for in-the-moment surveys, the average willingness to participate is similar, (only +4 percentage points [pp], significant at 5% level), supporting the initial hypothesis (*H2b*). Interestingly, the willingness to participate in in-the-moment surveys for the best scenario is significantly higher than for sharing geolocation (+7 pp), whereas for the worst scenario it is significantly lower (-3.2 pp), indicating that the precise combination of attributes offered to participants makes more difference in the former. In any case, these results seem to indicate that taking in-the-moment surveys in addition to sharing geolocation data does not reduce the willingness to participate, quite the contrary.

### 6.3. Comparing panelists with different characteristics

Next, to answer *RQ3*, the average willingness to participate was calculated for different groups of participants (see section 5.4.4.). Table 4 shows the average willingness to participate per covariate level and indicates when differences among each pair of levels of a covariate are significant.

**Table 4:** Willingness to participate per group of panelists.

Group	Covariate	Level	Avg. willingness (%)	
**Sociodemographic**	Gender	Male	47.8	^a^
		Female	42.3	^a^
	Age	18–34	50.9	^a^ ^b^
		35–54	45.4	^a^ ^c^
		55–74	38.8	^b^ ^c^
	Education	Low	47.7	^b^
		Mid	49.2	^c^
		High	38.9	^b^ ^c^
	Household size	Small	41.0	^b^
		Middle	42.9	^c^
		Large	51.4	^b^ ^c^
**“Big 5” personality traits **	Agreeableness	Neg.	35.0	^a^
		Posi.	48.3	^a^
	Consciousness	Neg.	40.5	^a^
		Pos.	45.0	^a^
	Extraversion	Neg.	32.3	^a^
		Pos.	48.2	^a^
	Stability	Neg.	40.4	^a^
		Pos.	47.7	^a^
	Openness	Neg.	39.6	^a^
		Pos.	48.5	^a^
**Attitudes/behaviors **	Survey privacy	Low	47.9	^a^
		High	21.8	^a^
	Survey safety	Low	63.3	^a^
		High	40.4	^a^
	Social media	Low	21.5	^a^
		High	60.2	^a^
	Installing apps	No	24.3	^a^
		Yes	55.6	^a^
	Google maps	No	21.5	^a^
		Yes	49.6	^a^
**Experience as panelists **	Surveys	Low	50.0	^a^ ^b^
		Mid	41.8	^a^ ^c^
		High	44.4	^b^ ^c^
	Metered data	No	40.0	^a^
		Yes	58.1	^a^

a b c indicate a significant difference (5%) between two levels of a covariate.

Regarding sociodemographic characteristics, higher willingness to participate is found for people who are male (support for *H3a*), young (support for *H3b*), mid-educated (no support for *H3c*) and living in large households (no support for *H3c*). Most differences among sociodemographic groups are significant but of limited size. The largest difference is found for age: the willingness of the oldest group is -12.1 pp compared to the youngest one.

The five personality traits studied are related to a significant positive effect on the willingness (support for *H3e* and *H3f*, but not for *H3g*, *H3h* and *H3i*). The size of the effects is moderate: extraversion is the trait influencing the willingness to participate the most (+15.9 pp) and consciousness the least (+4.5 pp).

As for attitudes/behaviors, panelists less concerned with survey privacy and safety, and frequently share contents in social medial, install apps and use Google Maps, are significantly more willing to participate (support for *H3j*, *H3k*, *H3l*, *H3m* and *H3n)*. The effect sizes are the largest among the covariates studied: -26.1 pp for survey privacy, -22.9 pp for survey safety, +38.7 pp for social media, +26.2 pp for installing apps and +28.1 pp for using Google Maps. Regarding the latter, two alternative variables to measure familiarity with geolocation-related apps’ features were also explored leading to similar conclusions: “sharing geolocation through WhatsApp” (+26.2 pp) and “allowing Google to show alerts based on your geolocation data” (+38.7 pp).

Finally, the expected positive effect of having more experience participating in panel surveys is not found when the number of past participations is considered for the entire panelists’ lifetime. However, a positive effect is found when this number is considered for the last three months only (+10.8 pp between low and high number of past participations, significant at 5%), which partially supports *H3o*. Moreover, as expected (*H3p*), metered panelists are significantly more willing to participate (+18.1%).

### 6.4. Main reasons to participate or not as stated by panelists

In order to answer *RQ4*, Table 5 shows the prevalence of the main reasons stated by panelists to participate or not in the two proposed geolocation-based activities.

**Table 5:** Main reasons to participate or not in geolocation-based research activities.

**Main reasons stated to participate**			
**Sharing geolocation (n = 520)**	**%**	**In-the-moment surveys (n = 593)**	**%**
Same reasons as for regular surveys	28.8	Incentive	26.7
Incentive	28.6	Same reasons as for regular surveys	21.0
Collaborate	19.1	Collaborate	13.9
Interest / curiosity	14.2	Interest / curiosity	12.3
Others	14.8	Others	17.4
**Main reasons stated to not participate**			
**Sharing geolocation (n = 388)**	**%**	**In-the-moment surveys (n = 284)**	**%**
Privacy	74.0	Privacy	76.1
Not willing to install an app	16.9	Not willing to install an app	17.4
Others	16.6	Lack of time	6.6
		Others	9.7

The results for both geolocation-based research activities are similar. When the participants were asked about the reasons for participating, one of the most mentioned answers was that they did not see any greater inconvenience in doing so compared to conventional panel surveys. Excluding this answer, as expected (*H4a*), the incentive is the most mentioned reason for both research activities. The remaining answers were not particularly related to geolocation-based research except interest in/curiosity for a new form of research. It is worth mentioning that several participants assumed that they would receive feedback about their data (e.g., frequent routes, average distance travelled), even if this was not indicated in the description of the activities. This suggests that such feedback, whenever possible, could be an effective motivation for some participants. This finding is in line with Struminskaya and colleagues [1], who found that respondents wanting to receive feedback in the form of summary reports have a significantly higher willingness to share mobile sensor data.

As for the reasons for not participating, privacy concerns (including lack of trust and safety issues) are mentioned by more than 70% of respondents for both activities (support for *H4b)*. The second most frequently mentioned reason is “not willing to install an app” with around 17% of mentions for the two activities. Grouping this answer with other problems related to the installation of an app (device performance, battery duration, insufficient memory, limited data plans and lack of technical skills), such mentions increase to around 24%. The negative effect of installing an app on the willingness to participate in research activities has been reported in the existing literature [40]. The remaining reasons for not participating are quite similar between activities, with one exception: lack of time was mentioned exclusively for in-the-moment surveys but only by 6.6% of the participants.

## 7. Discussion

### 7.1. Summary of main results

The average willingness to share geolocation data measured in this study is in line with previous literature (43.2%; *RQ1a*). Asking panelists to additionally participate in in-the-moment surveys does not substantially change such willingness (47.2%; *RQ1b*).

Moreover, results show that researchers can influence willingness through the decisions they make when planning the data collection: the different combinations of attribute levels studied can lead to a willingness that ranges from 37.6% to 50.1% for sharing geolocation data, and 34.4% to 57.1% for in-the-moment surveys (*RQ2*). Among the attributes considered, project duration is more relevant than incentive when it comes to sharing geolocation data, whereas when in-the-moment surveys are added, incentives become the most relevant attributes.

Regarding the direction of the effects of such attributes, panelists are more willing to participate when offered shorter projects, longer surveys up to 15 minutes, longer invitation lifetimes and higher levels of incentive. All these effects are in line with previous literature except for survey length. Moreover, the specific situation in which participants find themselves when they are invited to participate in in-the-moment surveys does not seem to affect their willingness substantially (at least among the five examples explored).

Regarding the differences among participants (*RQ3*), almost all the individuals’ characteristics assessed produced significant effects, not always in the expected direction. People who are males, younger, mid-educated and living in larger households are more willing to participate. Also those with positive values of the Big 5 personality traits, more experience completing panel surveys in the last 3 months, and who already share metered data are more willing to participate. However, the largest positive effects are found for panelists less concerned with survey privacy and safety, and panelists frequently sharing content on social medial, installing apps and using Google Maps.

Finally, when asked about reasons to participate or not (*RQ4*), incentive is the most frequently mentioned reason to participate and privacy to not participate, as expected. Furthermore, a considerable proportion of participants mentioned being interested in or feeling curious about geolocation-based research activities as reasons to participate.

### 7.2. Limitations

The above results must be considered carefully. First, stated willingness to participate is not actual participation. Ochoa and Revilla [11] describe the different ways in which both figures may differ. Although the difference has been found not to be large in sharing geolocation data activities (see section 2.1), it may be quite different for in-the-moment surveys due to practical issues that hardly can be foreseen by participants when declaring their stated willingness (e.g., not seeing the invitation in time [10]). Further experimental research is needed on this specific issue. Second, I focused on members of an opt-in online panel, assuming that this will be the source of participants used to develop in-the-moment surveys in most cases. Further research is needed to assess whether similar results are found for other kind of samples (e.g., probabilistic samples or specific communities of people). Additionally, I used one particular opt-in online panel (Netquest) in one country (Spain). Other panels and countries may produce different results. Third, the method used (CBC) is sensitive to the selection of the attributes and levels. Although this limitation exists in almost any alternative method, CBC is particularly sensitive to the levels chosen to cover each attribute. Different selections may produce different evaluations of the attributes’ importance and, potentially, different levels of willingness to participate.

### 7.3. Practical implications

All in all, these results suggest that combining geolocation data and in-the-moment surveys does not raise additional concerns compared to only sharing geolocation data in terms of willingness to participate. The inconveniences of in-the-moment surveys seem to be irrelevant compared to the concerns related to sharing geolocation (i.e., privacy, installation of an app). However, experimental research is needed to reveal practical difficulties that should be addressed to develop in-the-moment surveys, as well as their actual value.

The results of this study can guide researchers to (1) adjust the conditions offered to online panelists when asked to share geolocation data, and (2) design strategies to develop in-the-moment surveys. Such results highlight the relevance of the project duration, which forces researchers to face a difficult trade-off when planning to use in-the-moment surveys: detecting events of interest may require sharing geolocation data for long periods (especially for occasional events such as visiting a hospital), but long periods negatively affect participants’ willingness. Researchers may consider an alternative approach: shortening the project duration and increasing the number of individuals sharing geolocation data. Although increasing the sample size is often a challenge, the increased response rate can be worth it. Interestingly, results of this study suggest a third option: participants may tolerate sharing geolocation data for an undefined period (willingness equivalent to requesting data for three months) if they can give up at any time. However, further research is needed to assess which strategy works best in practice.

Besides the relevance of the project duration, incentives are still key to motivate panelists to participate in in-the-moment surveys, especially considering the inconvenience of having to participate in the moment. In that sense, giving participants feedback about their mobility (which may require storing the collected geolocation data even for in-the-moment surveys) could increase their willingness to participate. Further research is needed on this topic as well.

Regarding sample composition, this research has found differences among participants in terms of willingness to participate. Although sociodemographic variables are the most frequently used to control quotas in data collection developed on online panels, researchers should consider controlling for other attitudinal/behavioral variables that seem to produce larger differences among participants. To what extent such differences may cause bias on the specific variable of interest must be addressed, attending to the particularities of each study.

To conclude, this research suggests that in-the-moment surveys triggered by geolocation data should be feasible if developed on samples drawn from online panels, at least from the standpoint of potential participants’ willingness to participate. Real in-the-moment surveys are needed to prove the actual value of this method to improve data quality.
